# A novel and safe technique in closed tube thoracostomy

**DOI:** 10.1186/1749-8090-5-21

**Published:** 2010-04-06

**Authors:** Koray Dural, Gultekin Gulbahar, Bulent Kocer, Unal Sakinci

**Affiliations:** 1 Numune Teaching and Research Hospital, Division of Thoracic Surgery, Ankara, Turkey; 2 Dr. Nafiz Korez Sincan City Hospital, Division of Thoracic Surgery, Ankara, Turkey

## Abstract

**Background:**

Tube thoracostomy (TT) is the most commonly performed surgical procedure in thoracic surgery clinics. The procedure might have to be repeated due to ineffective drainage in patients with tube malposition (TM), in whom the drain is not directed to the apex or located in the fissure. Trocar technique, which is used to prevent TM, is not recommended because of its potential for severe complications.

**Methods:**

The study involved 180 patients who required TT application for any etiology within one year. The patients were divided into two groups as Group A, who had undergone classical surgical technique (n = 90) and Group B, who had undergone a combination of surgery and trocar techniques (n = 90). The groups were compared for TM, the effect of TM on the drain removal, and other insertion related complications.

**Results:**

In Group A, 23 patients had TM, 4 of whom developed associated ineffective drainage, while the patients in Group B had no insertion related complications (p = 0.001). The mean drain removal time of the patients with TM was 5 ± 2.25 days. In the patients who did not develop TM, it was 3.39 ± 1.18 days (p = 0.001).

**Conclusions:**

The modified combination technique is a reliable method in preventing TM and its potential complications.

## Introduction

TT is a standard and generally reliable method in the management of pathologies responsible for accumulation in the pleural space [1]. The two most commonly used methods in the thoracic surgery clinics are surgery and trocar technique. Because the incidence rate of pulmonary parenchyma and intrathoracic organ injury is increased by trocar technique procedures, it is now used in very few centers. This study aimed to investigate the effects of combined modified technique that involves surgery and trocar technique on tube malposition (TM) and other potential complications.

## Materials and methods

### Patients

This randomized, prospective study involved 180 patients who required TT for various etiologies between 2006 and 2007. The detection of the type of method to be used for the allocated patients were determined by using the prepared bloc randomization lists before the study. After receiving the statement of patient consent from all patients, the patients were evaluated in two groups as those who were applied surgical technique (Group A) and those who were applied combined modified technique (Group B). The presence of severe pleural adhesion was considered a contraindication for modified technique and these patients were applied surgical technique. On the chest computer tomography (CT), fissural, parenchymal, and extrathoracic location of the drain and angulation of the drain in the interpleural space were considered TM. The cases confirmed as TM depending on poorly air and fluid drainage, and because of that the pulmonary expansion could not be possible adopted as ineffective drainage. The groups were compared for TM occurrence, the effect of TM on the time of drain removal, and other tube thoracostomy complications.

### Diagnostic method

The patients who were scheduled for TT application for various indications were evaluated through AP and lateral radiographs before and after the procedure. The patients with suspected TM or severe complications were evaluated through thorax CT.

### Surgical technique

Surgical TT was performed in all the patients. After local anesthesia was achieved with lidocaine HCl, TT was performed through 5^th^, 6^th^, or 7^th ^intercostal space, or at the anterior or mid-axillary level depending on the etiology. In this technique, the stages of TT application are as follows: after sterilization of the area with betadine solution, an incision of nearly 2 cm is made on the proper location where it is parallel to the upper margin of the lower rib. Following blunt dissection with dissection clamp, the pleural space is penetrated. Any parenchymal adhesions, if present, are eliminated through finger exploration. A thorax drain of proper size (28 or 32F) for the indication is placed into the pleural space with the help of a macro clamp and connected to an underwater drainage system. Finally, a U-suture is made for use in drain fixation and removal.

### Modified combined technique TT

The patients were prepared as in the surgical technique and were performed local anesthesia. An incision of nearly 2 cm was made at the proper location. In trocar technique, after penetrating into the pleural space, the trocar and drain together are advanced towards the apex by using force. This technique was modified and started as in the surgical technique. After penetrating the pleural space with blunt dissection, the adhesions in the pleural space were explored using a finger. Then, thoracic trocar and 28-32F XRO translucent PVC drain (Vygon^®^) was advanced towards the pleural space and directed to the apex. After the combination was advanced about 15 cm, the trocar was withdrawn about 10 cm, and thus, the free end of the drain was advanced until it reached and was placed in the apex. Then, the trocar was withdrawn until the end of the drain that was outside the thorax. Upon clamping the drain on its proximal, the trocar was completely withdrawn and the drain was connected to the underwater drainage system. After the drain was fixed, the procedure was completed.

### Statistical Method

Chi-square test was used for significance and p < 0.05 was considered statistically significant.

## Results

Group A comprised 87 male patients (96. 7%) and 3 female patients. The mean age of the patients in Group A was 34.70 years. Group B comprised 81 male patients (90%) and 9 female patients. The mean age of the patients in Group B was 35.75 years.

The most common etiology in both groups was stab wound injuries (Table 1).

**Table 1:** The distribution of the patients in both groups according to their etiologies.

Etiology	Group A		Group B	
	
	n	%	n	%
Stab wound injuries	30	33.3	28	31.1

Firearm injury	9	10	6	6.7

Spontaneous pneumothorax	28	31.1	27	30

Motor vehicle injuries	9	10	8	8.9

Pedestrian injuries	5	5.6	5	5.6

Assault	3	3.3	3	3.3

Fall	4	4.5	11	12.2

Pleural effusions	1	1.1	1	1.1

Empyema	1	1.1	1	1.1

**TOTAL**	**90**	**100**	**90**	**100**

The differance between clinical characteristics of two groups were insignificant, statistically (p > 0.05; Chi-Square test).

The most common late-stage complication associated with TT was prolonged air leak (Table 2).

**Table 2:** The distribution of the patients in both groups according to late-stage complications.

Complications	Group A		Group B	
	
	n	%	N	%
Prolonged air leak	6	6.7	4	4.4

Ineffective drainage	4	4.4	0	0

Replaced drain	0	0	2	2.2

Complications associated with drain removal	2	2.2	1	1.1

**TOTAL**	**12**	**13.3**	**7**	**7.8**

**Figure 1:**
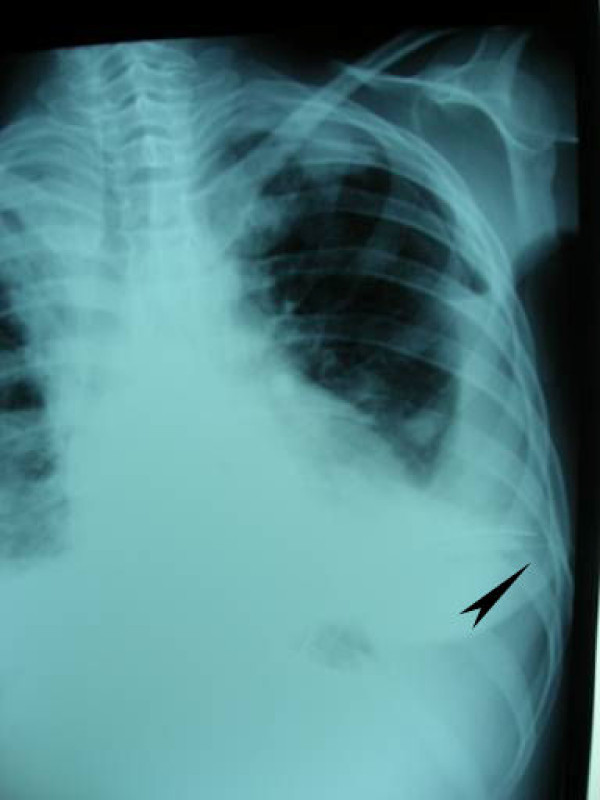
**The X-ray image of the patient in whom tube thoracostomy was performed on the left side and the drain was directed to the diaphragm rather than the apex (tube malposition)**. Despite the drain, hydropneumothorax is observed.

**Figure 2:**
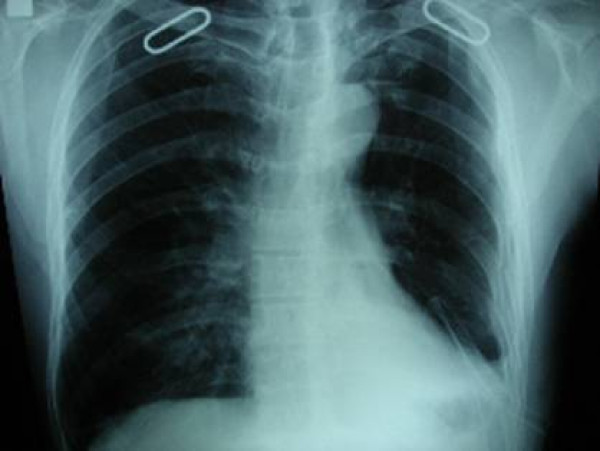
**X-ray image of the other tube malposition patient**. Despite the drain, retained haemothorax is observed.

In Group A, 23 (25.5%) patients developed TM, and in 4 (4.4%) of these patients, TM led to ineffective drainage (Figure 1, Figure 2). In Group B, however, no technical complications occurred (p = 0.001).

In the patients with no TM, the mean drain removal time was 3.39 ± 1.18 days, while in the patients with TM, the mean drain removal time was 5 ± 2.25 days (p = 0.001).

## Discussion

TT is the most commonly performed surgical procedure in thoracic surgery clinics [1,2]. In experienced hands, its complication rate is low and can be used safely [3]. One of the important factors affecting the complication rates of TT is the method of TT used. Severe complications have been reported particularly during TT procedure; thus, in many clinics, trocar technique is no longer used [4-7]. In this method, application of drain without finger exploration can cause pulmonary parenchymal injuries because of the impossibility of determination of the pleural adhesions. Also pulmonary, cardiac, esophageal and main vascular injuries may occur by trochar that has a sharp point en route pleural space. In the other hand failing the trochar assistance; it could not be possible to place the drain into the pleural space without TM formation. This identified modified combined technique has a purpose to take advantages of the other two techniques.

When the drain is not directed towards the apex in the pleural space, it is termed as TM. This complication occurs in 4 locations: intraparenchymal, fissural, extrathoracic locations, and angulation of the drain in the pleural space. Although TM usually occurs in urgent TT procedures, it may also be associated with the method of TT used. In a study where TM was defined in fissural or parenchymal location, trocar technique was shown to increase the risk of TM [8].

On the other hand, angulation of the drain in surgical technique is not a rare occasion. In such cases, poor drainage may be associated with the angulation point and the degree of angulation of the drain because angulation often reduces the diameter of the lumen. This may result in failure in effective drainage depending on the diameter of the parenchymal defect particularly in pneumothorax. Some authors recommend the withdrawal and reinsertion of the drain in case of TM, while others suggest keeping the drain in its place if effective drainage is achieved [9]. Although leaving the drain at its location even when TM is determined seems plausible, perhaps the only exception is intraparenchymal location of TM. This may cause prolonged air leak and its potential complications. Moreover, after the drain is removed, the resultant air leak from the defect that is caused by the drain may lead to pneumothorax or hemothorax or both.

With the use of the method described here, which consists of a combination of modified trocar and surgical TT techniques, we aimed to reduce the incidence of intrapleural angulations, which are commonly observed in surgical technique, and intraparenchymal-fissural locations, which are common with trocar technique, as well as pulmonary and cardiovascular injury. None of the patients involved in the study was performed trocar technique TT due to high complication rates. The comparison of the patients who were performed classical surgical technique and the patients who were performed modified combined technique revealed significant differences in favor of the group that was applied modified combined technique with respect to TM and air leak.

## Conclusion

The results of this study have shown that modified combined technique can be used safely in thoracic surgery clinics because it reduces the incidence rates of complications and TM, is easy to perform, and has positive effects on air leak and hospitalization time.

## Competing interests

The authors declare that they have no competing interests.

## Authors' contributions

This report reflects the opinion of the authors and does not represent the official position of any institution or sponsor. The contributions of each of the authors were as follows:

KD and GG were responsible for reviewing previous research, journal handsearching, drafting report. BK was responsible for provision of published trial bibliographies, preparing photographs. GG was responsible for quality checking, coding and classification, data processing. US was responsible for project coordination.

All authors have read and approved the final manuscript.
